# The Beer Bill

**Published:** 1901-04-06

**Authors:** 


### The Hospital

A Journal of

The Medical Sciences and Hospital Administration.

Vol. XXX.—No. 758] Edited by Sir Henry Burdett, K.C.B., and Solomon C. Smith, M.D., M.R.C.P. [April 6, 1901.

### The Beer Bill.

The House of Commons devoted nearly five hours of Wednesday in last week, nominally to the discussion of a "Beer Bill" introduced by the honourable member for Peterborough, but really to a demonstration of the unfitness of a popular assembly to decide any question in which the teachings of science require to be applied by the light of common sense. Proposals for legislation which it would be complimentary to designate merely as absurd were carried through a second reading by a majority of 112, and with the less excuse inasmuch as Sir Michael Foster, in an admirable and lucid maiden speech, clearly demonstrated the impossibility of putting them into practice, and their inutility even if the impossibility could be overcome. The fact that a single firm of manufacturers of sulphuric acid had for some months supplied a product contaminated with arsenic to a single firm of manufacturers of glucose and invert sugar, whose wares, extensively employed by brewers in certain districts of England, had been productive of an enormous amount of chronic arsenical poisoning, which was arrested as soon as its character and causes were discovered, was made the occasion for an attempt to prohibit the use of artificial sources of alcohol, or at least to enact that there should be a something to be called "malt beer," into the composition of which these artificial sources were not to enter, but which was to be made from malt and hops alone, and to be supplied to all customers who demanded it. The Bill would permit brewers to put anything they liked into a preparation which was to be distinguished from the former by the description "part malt beer," and which might be strengthened or flavoured in any way calculated to suit either the taste of the consumer or the pocket of the manufacturer. Sir Michael Foster explained that there was no possible way of discovering, in the case of any sample of beer, whether its alcohol had been derived entirely from malt or entirely from glucose or invert sugar, or from an admixture of these substances; and hence that the proposed character of "malt beer" could only be secured by continued inspection of breweries during the whole progress of the manufacture. He explained, moreover, that glucose and invert sugar were not employed so much as substitutes for malt as in addition to it, or as a means of introducing a larger amount of alcohol-producing material than the malt itself would contain, and that what is required for the protection of the public is, not the prohibition of good material, but the better enforcement of existing laws against adulteration or against the introduction into substances sold under a specific name of other materials foreign to their nature. The House applauded, but the voting did not appear to be influenced, and one honourable member has since written to the Press to say that his vote was given in the hope that the Bill, if passed into law, would in some way or other be beneficial to the agricultural interest. How the benefit was to be afforded he did not say, and probably did not very clearly realise.

It may not unreasonably be hoped that reflection will bring wiser counsels into operation, and that Mr. Purvis's Bill, by some contrivance or other, will be shipwrecked before it reaches the Committee stage in the House of Commons, or will at all events be suffered to die a natural death in the Lords. However this may be, it is humiliating in the twentieth century to find the oldest legislative assembly in the world dealing in this merely impulsive fashion with an entirely technical subject. On any question which really and truly affects the public health it is only too difficult to obtain effective legislation, and, as Sir Michael Foster pointed out, the laws which exist are very imperfectly administered. Of this the arsenical poisoning itself furnishes only too flagrant an example. Dr. Tattersall, the Medical Officer of Health to the Borough of Salford, in a very excellent report on the whole subject has pointed out that, although a large number of people were killed, and a vast number more or less seriously affected, from the beginning of June, the numbers increasing steadily up to the end of November in Manchester, Liverpool, Salford, and many other towns, it never seems to have occurred to anyone that it was worth while to put the machinery of an official enquiry into the cause of all this sickness into motion. There would have been no difficulty in doing so, for, as Dr. Tattersall farther shows, there is an Order of the Local Government Board, issued in

1879, requiring lists of cases of new sickness treated by Poor Law medical officers to be sent regularly to the Medical Officer of Health. If this Order, which appears to have fallen into complete desuetude, had been effectively carried out, attention must have been directed to the arsenical poisoning at a much earlier period, and many lives would have been saved. It should be supplemented, as a matter of course, by some adequate provision for investigating the nature and causes of any exceptional forms of illness to which it may be made the means of directing attention.

### The Census.

Once again we have undergone the process of numbering the people, and in due course may expect to ascertain how far the guesses at truth made by medical officers of health as to the population of their districts are correct. As we hardly need say, all the superstructure which goes by the name of vital statistics is founded upon the information given by the census in regard to the number, age, sex, condition, and occupation of the people. It is on the statements made in the census papers as to these primary details that depends all our knowledge as to birth and death rates, as to the comparative mortality of town and country, as to the healthiness or the opposite of the various trades, and as to the gradual movements of the population from place to place. Statistical investigations are always difficult enough, even when the facts are clear, but vital statistics are always complicated by the question of the reliability of the individual facts upon which they are founded. It is probably for this reason, and partly also in order that the statistics of one decennium may be the more readily compared with those collected on other occasions, that the number of the questions on the census papers is comparatively small. One cannot but feel, however, that if the census is to give any complete information as to the social or sanitary progress of the people, as distinct from what one may call interesting information, inquiry might well have been made in regard to many matters which have been left quite untouched. Putting on one side questions of number, sex, and age, which of course are the basis of everything, one of the most important inquiries made in the census papers which were filled up last Monday was that in the little square in the left-hand corner in which was to be stated the number of rooms occupied by the persons referred to in case they should occupy altogether less than five rooms. It is from the statements made in this little square that we shall be able to ascertain the condition of the people as to overcrowding, and to compare this with their state in that respect ten years ago. Much as the rest of the paper will tell as to growth and distribution of the population, and as to age, condition, and occupation, nothing will so markedly tell about prosperity and happiness or their opposites as the indications given in this little square which show the proportion of the population who find themselves compelled to pass their home-life crowded together into a small number of rooms. If the country shows that it has made progress in numbers, in wealth, and in longevity, some may say that all is well. But if it has progressed at the same time in overcrowding, and if the census should show that a larger proportion of the people than before are squeezed into a smaller home-space, no one who cares for the health conditions and the stamina of the race can be content.

### The Eyesight of School Children.

In an address recently delivered before a Conference of Teachers at the Durham College of Science Dr. Thomas Oliver pointed out the importance of examining the eyesight of school children, and of providing them with the treatment necessary for preventing their school life having a permanently injurious effect upon their vision. Obvious as is the desirability of every elementary school having attached to it a fully qualified medical officer, the question of expense has always stood in the way, and school boards have dealt with the medical treatment of children who are sick much in the same way as they have with the provision of meals for those who are hungry, maintaining—and apparently justly maintaining—that their work is only with education, and that all such matters as food, medical treatment, clothing, &c., however great their urgency, are outside the limits of their work, and must be undertaken by other agencies. Admitting all this to the full, we think with Dr. Oliver that the question of eyesight comes under a different category, in that the defects of vision, which tell so seriously upon the future usefulness of these school children, are in many cases the direct product of the education which they are by law compelled to undergo. "Admitting," says Dr. Oliver, "that a certain degree of long-sightedness is the natural condition of the infant's eye, and that this is corrected as development proceeds, short-sightedness, on the other hand, is not a hereditary defect, but one for which school life is very largely, if not altogether, responsible." "Myopia is the bane of modern school life." "In every country in the civilised world the number of short-sighted people is increasing in proportion to the amount of eye-work undertaken by children in the schools." If this is to be taken as a full presentment of the state of affairs it is clear that the responsibility of educational authorities in regard to eye troubles stands on a very different footing from that in regard to other disorders. The eye troubles can in many cases be traced directly to the education, and that being so the question is whether the compelling authority should not provide the means of preventing what otherwise must be regarded as a considerable drawback to the undoubted advantages of our educational system. Dr. Oliver says: "If, while we are educating children in the hope of fitting them for their life's work . . . we are ultimately unfitting them, and creating a large number of blind people requiring to be maintained either at the expense of charity or the public, then money is simply being taken, not out of one pocket only, but out of both the pockets of the ratepayer."

### The Conservative Crofter.

A recent incident in the history of the Scotch Congested Districts Board may shed some light, not only on the difficulties of dealing in a satisfactory way with the land question, but on the problems of poverty generally. A group of crofters at Sconser in the island of Skye are at starvation point. The holdings they occupy are of the poorest, a thin sprinkling of earth on a rocky subsoil, and their numbers are fast outrunning their means of subsistence, even of the most meagre subsistence. In pity for their condition the Congested Districts Board has offered to remove the little colony to better land not many miles away, and to assist them with new seeds and in other ways to get their new holdings in order—all this is to be done without cost to themselves—and the Sconser crofters refuse to go! The stupidity shown by these people makes one feel that sometimes eviction itself might be the truest kindness. This at least we should infer from the records of a Scottish colony of the crofter class which has been settled by the Crofters' Colonisation Commissioners at a township in Manitoba called Killarney. The Commissioners do not regard these crofters as ideal colonists, but at least they have in the twelve years that have passed since they were settled there brought nearly 4,000 acres of land under cultivation. There are six homesteads and 260 persons, and both their wheat crop and their holding of live stock are steadily increasing. In short, those who have industry and energy are doing well, and there seems no doubt that those who have stuck to their holdings are incomparably better off than their compatriots in the Hebrides. And yet their Sconser brethren will not move even a few miles away from their old home to obtain better conditions. But this lack of energy and initiative is the great fault of the ignorant. Where and as they were born, there and so they will die, in their native croft, or bog, or slum. This is one of the greatest difficulties in the way of the sanitary, as well as of the social, reformer. Contentment with their lot and willingness to bear their misfortunes without a murmur are by some regarded as virtues, but they are terrible obstacles to sanitary progress.

### Notification and Libel.

A matter of some interest arises in reference to a decision lately given in an American court. It has there been decided that "to falsely publish of one that he has leprosy is libellous, since the disease, whether properly or not, is still commonly regarded as infectious or contagious, and as tending to cause the sufferer to be shunned or excluded from society." Now, with the gradual spread of the opinion that tuberculosis is in the same way infectious or contagious, one can hardly help seeing that there is also gradually growing up among large numbers of thoughtful and intelligent people a very definite dislike to coming into personal relations with consumptives. However carefully those who preach the crusade against consumption may guard their statements as to the infectious nature of the disease with other statements to the effect that by proper care all danger of infection can be avoided, most people will, and, in fact do, say that if such precautions are necessary the safest plan is to give the consumptive a wide berth. It is beginning to be the case, and as the text of the crusade is driven home it will be the case more and more, that the fact of being consumptive causes a man to be "shunned or excluded from society," and when we find sanitary authorities inviting us to establish a system of voluntary notification of consumption, as is now being done in various towns, it is worth our while to pause a moment and ask what remedies at law a man might have against us if, in consequence of our notification, he were to lose his employment or otherwise suffer inconvenience, especially if he were at the same time able to offer medical evidence, perhaps quite as good as our own in the eyes of the court, to the effect that he was not consumptive at all; certainly not an impossible event in view of the tendency of doctors to disagree. We may be very sure that "privilege" would not cover a communication which the practitioner is under no statutory obligation to make.

### Smoke Abatement.

The annual report of the Coal Smoke Abatement Society contains matter of much interest, partly pertaining to the abatement of smoke, partly throwing light—if smoke can ever be said to throw light—upon the subject of how we are governed. It appears that since the formation of the society, in March 1899, no fewer than 2,700 observations have been made by the society's inspector on smoking chimneys in various parts of London; that offences have been proved in 162 cases; and that penalties have been inflicted amounting altogether to the large sum of £750 All of this is so much to the good, and so far as as it goes will tend to give to Londoners a little more of that pure air and sunshine which is so essential to their health. The interesting point, however, about the whole thing is that all this work ought to have been unnecessary, that all this inspection of chimneys and all this prosecution of evil doers ought to have been undertaken by the municipal authorities—the vestries that were, the boroughs that are—and that if the duty which properly lies upon these authorities had been performed with reasonable efficiency, it would never have been necessary for a private body to take action at all. For those who profess to think that as a result of the gradual municipalisation of everything, a time will come when whatever is good for us will be done by "the authorities" and private initiative will become a thing of the past, it may be a useful lesson to consider the amount of work which the Mansion House Council on the Dwellings of the Poor, and the Smoke Abatement Society find to do, for it may safely be said that the mass of the work of both these organisations consists of what has been scamped or left entirely undone by the local authorities which are appointed for the very purpose of doing it.

### Coroners and the General Public.

Medical men so often suffer from the tyranny of the coroner that it is with a certain degree of satisfaction that we now and again see the general public treated to a taste of that sweet and pleasant humour which coroners find so appropriate to the dignity of their Courts. At an inquest held at Mortlake last Saturday the question arose as to whether the proceedings were to be open to the public. According to the report of the proceedings in the daily papers, Mr. Reginald Schomberg, a barrister and an old inhabitant, asked the coroner to hear him, contending that the public were being excluded although it was a public Court. To this the coroner is reported to have answered that there were 16 gentlemen on the jury, and members of the Press in Court, and "if they did not represent the public he did not know who did." Mr. Schomberg, however, protested that there was plenty of room, and that there were a number of the general public in waiting for admission, to which he received the polite retort from the coroner that "in his opinion the general public were a general nuisance." Clearly this good coroner was a trifle puffed up with office, for he went on to say that "if he and his jury could not look after the level crossings he did not know who could." Whether the coroner did in fact make a note that the general public were excluded, as he was requested to do by Mr. Schomberg, we do not know, but in any case it is important that such a note should be made. We have every respect for those diligent and long-suffering members of the Press who attend inquests, and with commendable zeal endeavour to pick out "racy bits" to relieve the tedium of their papers, but we can hardly admit that they represent the general public.

